# Exciton–polaritons in van der Waals heterostructures embedded in tunable microcavities

**DOI:** 10.1038/ncomms9579

**Published:** 2015-10-08

**Authors:** S. Dufferwiel, S. Schwarz, F. Withers, A. A. P. Trichet, F. Li, M. Sich, O. Del Pozo-Zamudio, C. Clark, A. Nalitov, D. D. Solnyshkov, G. Malpuech, K. S. Novoselov, J. M. Smith, M. S. Skolnick, D. N. Krizhanovskii, A. I. Tartakovskii

**Affiliations:** 1 Department of Physics and Astronomy, University of Sheffield, Sheffield S3 7RH, UK; 2 School of Physics and Astronomy, University of Manchester, Manchester M13 9PL, UK; 3 Department of Materials, University of Oxford, Parks Road, Oxford OX1 3PH, UK; 4 Helia Photonics, Livingston EH54 7EJ, UK; 5 Institut Pascal, Blaise Pascal University, 24 avenue des Landais, 63177 Aubiére, France; 6 Physics and Astronomy, University of Southampton, Highfield, Southampton, SO17 1BJ, UK

## Abstract

Layered materials can be assembled vertically to fabricate a new class of van der Waals heterostructures a few atomic layers thick, compatible with a wide range of substrates and optoelectronic device geometries, enabling new strategies for control of light–matter coupling. Here, we incorporate molybdenum diselenide/hexagonal boron nitride (MoSe_2_/hBN) quantum wells in a tunable optical microcavity. Part-light–part-matter polariton eigenstates are observed as a result of the strong coupling between MoSe_2_ excitons and cavity photons, evidenced from a clear anticrossing between the neutral exciton and the cavity modes with a splitting of 20 meV for a single MoSe_2_ monolayer, enhanced to 29 meV in MoSe_2_/hBN/MoSe_2_ double-quantum wells. The splitting at resonance provides an estimate of the exciton radiative lifetime of 0.4 ps. Our results pave the way for room-temperature polaritonic devices based on multiple-quantum-well van der Waals heterostructures, where polariton condensation and electrical polariton injection through the incorporation of graphene contacts may be realized.

## Abstract (web summary)

Polaritons are exciton–photon hybrid particles that are created when the interaction between light and matter is strong enough. Here, the authors create polaritons in molybdenum diselenide/boron nitride quantum wells by enhancing the light–matter coupling using a tunable optical cavity.

## Graphical abstract

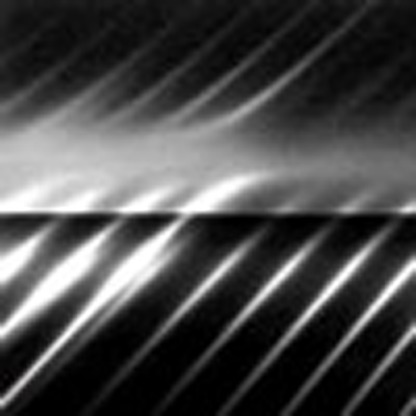

Recently, the fabrication of vertical assemblies of two-dimensional (2D) materials has become possible providing novel types of heterostructures with controlled and tunable properties^1^,^2^,^3^. The weak interlayer bonding allows a variety of 2D layers with different lattice constants to be stacked on top of one another, creating artificial materials with new material characteristics^3^. The potential applications of these van der Waals (VDW) heterostructures are further widened through the incorporation of semiconducting transition metal dichalcogenide (TMDC) monolayers^4^,^5^. Unlike their bulk form, monolayers of semiconducting TMDCs such as WS_2_, MoS_2_, WSe_2_ and MoSe_2_ have direct bandgaps^5^,^6^. They exhibit pronounced exciton resonances at room temperature owing to the exceptionally high exciton-binding energies of a few 100 meV (refs 7, 8, 9, 10, 11), as well as display coupled spin and valley degrees of freedom^12^,^13^,^14^. Recently, electroluminescence from lateral p–n junctions based on hBN–WSe_2_ heterostructures^15^ has been demonstrated, and stacking of TMDC layers has been used to demonstrate the photovoltaic effect^16^, tunnel diodes and transistors^17^ and the formation of long-lived interlayer excitons^18^. In more complex heterostructures incorporating hBN barriers and graphene electrodes, efficient electroluminescence was observed from WS_2_ and MoS_2_ monolayers under vertical current injection^19^.

The integration of TMDC heterostructures in optical microcavities is also an attractive alternative to systems where the strong light–matter interaction has been studied previously. In this regime, formation of part-light–part-matter quasi-particles called exciton–polaritons is observed. The study of exciton–polaritons has revealed a wealth of rich phenomena such as Bose–Einstein condensation in the solid state^20^, polariton superfluidity^21^, as well as room-temperature polariton lasing in the ultraviolet and blue spectral regions using wide bandgap materials such as GaN (refs 22, 23) and ZnO (refs 24, 25) or organic materials^26^,^27^,^28^. Very large exciton-binding energies and sharp exciton resonances in TMDCs are important prerequisites for observation of the strong coupling in these materials. The exciton properties can further be tailored by combining a wide variety of 2D crystals in heterostructures. Observation of room-temperature excitons in TMDCs, combined with the recently demonstrated good electroluminescence of VDW heterostructures^19^, lays the foundation for the development of low-threshold electrically pumped polariton lasers operating in the visible and near infrared. These devices can be easily incorporated onto a wide range of substrates allowing the development of hybrid TMDC/III-V microcavity structures as well as electrically driven polariton devices with vertical current injection using graphene contacts.

In this work, we report on the observation of the strong-coupling regime in TMDC heterostructures, underpinning such polariton-based technologies. We place MoSe_2_/hBN heterostructures in open tunable cavities having high reflectivity dielectric mirrors with adjustable separation^29^,^30^. In contrast to previous experiments, where modification of the emission pattern and radiative recombination rate of 2D films coupled to cavities was observed^30^,^31^,^32^, as well as a recent reports on lasing in photonic crystal nanocavities and in microdisks^33^,^34^, we present conclusive evidence for the strong light–matter interaction regime and the formation of part-light–part-matter polariton eigenstates. This regime is observed when the cyclic emission and reabsorption of light inside a microcavity occurs on a timescale faster than the exciton and photon dissipation rates, a regime favoured by the large oscillator strength of the direct-bandgap optical transition in TMDC monolayers^11^,^35^,^36^. Here we evidence strong coupling in reflectivity and photoluminescence (PL) through the anticrossing of the tunable cavity mode energy and the MoSe_2_ exciton energy showing the formation of upper and lower polariton branches (UPB and LPB). A large Rabi splitting of 20 meV is observed for a heterostructure containing a single MoSe_2_ monolayer, whereas this splitting is increased to 29 meV for a multiple-quantum-well structure with two MoSe_2_ monolayers separated by a hBN layer. From the coupling strength, we extract a radiative exciton lifetime of 0.4 ps corresponding to a homogeneous linewidth of 1.6 meV. In contrast to previous work in TMDC microcavities, where the observed spectral features are poorly resolved preventing an unambiguous claim of strong coupling^37^, we show fully resolved polariton branches with a Rabi splitting that significantly exceeds the polariton linewidths. We also observe a notable difference between the exciton–photon coupling for the neutral (X^0^) and negatively charged (X^−^) excitons, with weak coupling observed for X^−^, since the estimated coupling strength of 8.2 meV is less than its linewidth, preventing charged polariton branches from being resolved. Moreover, the large exciton-binding energy of TMDCs allows the observation of a narrow UPB with comparable intensity to the lower polariton at resonance. This arises due to the significant separation of the electron–hole continuum from the upper polariton states, potentially opening a new regime of polariton physics.

## Results

### VDW heterostructures embedded in a tunable microcavity

**Figure 1:**
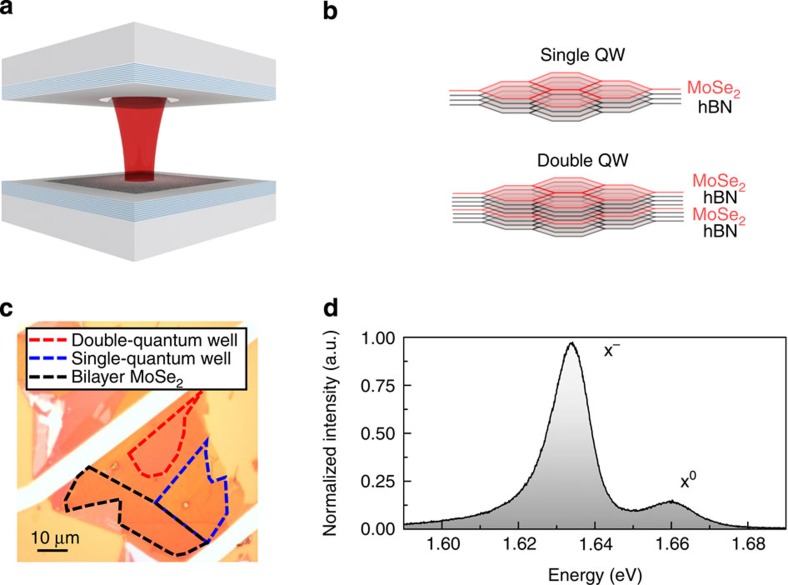
MoSe_2_ heterostructures embedded in a tunable open-access microcavity. (**a**) Schematic of the tunable hemispherical cavity with an embedded MoSe_2_ heterostructure. (**b**) Schematic of the single- and double-QW heterostructures. (**c**) Optical image of the MoSe_2_ heterostructure where the single- and double-QW areas are marked by the blue and red boxes, respectively. A bilayer MoSe_2_ region is marked by the black lines. The structure is fabricated on the surface of the planar DBR at an electric-field antinode of the formed microcavity. (**d**) A PL spectrum of the single-well monolayer of the MoSe_2_ heterostructure at 4.2 K exhibiting two characteristic peaks attributed to a neutral (X^0^) and charged (X^−^) exciton with a measured linewidth of 11 meV for X^0^ and 15 meV for X^−^.

The tunable microcavity is formed by one planar and one concave dielectric-distributed Bragg reflector (DBR)^38^. A schematic of the formed microcavity is shown in Fig. 1a where two nanopositioner stacks allow the independent positioning of the two DBRs and the cavity mode resonances can be tuned *in situ* through control of the mirror separation. The heterostructure is fabricated through standard mechanical exfoliation and then transferred to the surface of the planar DBR, at an electric-field antinode of the formed microcavity. The heterostructure consists of different areas corresponding to three different active regions: a single-quantum-well (QW) region and a double-QW region schematically shown in Fig. 1b, and a bilayer MoSe_2_ region. The single-QW region consists of a single monolayer sheet of MoSe_2_ placed on a 3-nm-thick sheet of hBN as outlined by the blue dashed lines in the optical image in Fig. 1c. The double-QW structure consists of two monolayer sheets of MoSe_2_, separated by a 3-nm film of hBN, placed on a thin sheet of hBN outlined by the red dashed lines in Fig. 1c. In addition, an area of a bilayer MoSe_2_ is enclosed within the black dashed lines. The low-temperature PL spectrum from a single monolayer of MoSe_2_ is shown in Fig. 1d. The spectrum consists of a neutral exciton X^0^ and a negatively charged trion X^−^ with linewidths of 11 meV and 15 meV, respectively^39^.

### Strong exciton–photon coupling

**Figure 2:**
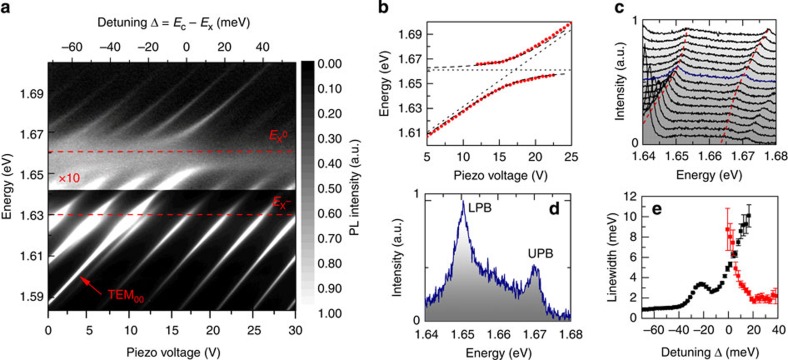
Observation of strong exciton–photon coupling in a MoSe_2_ single-QW heterostructure. (**a**) A clear anticrossing in PL is observed between the discrete cavity mode energies and the neutral exciton energy at 4.2 K. The longitudinal resonance is labelled TEM_00_. (**b**) The upper and lower polariton branches are fitted for the longitudinal mode with a vacuum Rabi splitting of 20 meV. (**c**) PL spectra of the longitudinal mode at various exciton–photon detunings from Δ=−16 meV (bottom) to Δ=+12 meV (top). (**d**) The PL spectrum on resonance shows the UPB and LPB well resolved. (**e**) The linewidth of the LPB and UPB as a function of detuning (black and red symbols, respectively). On resonance, the linewidth for LPB and UPB is around 4.8 and 8.5 meV, respectively. At a negative detuning of Δ=−25 meV, the LPB is broadened due to weak coupling with the charged exciton state.

The open-cavity system allows spatial xyz-positioning of the two mirrors independently. As such, any area of the MoSe_2_ heterostructure on the planar DBR can be placed in the optical path and a microcavity formed with the selected area as the active region. The micron-sized Gaussian beam waist on the planar mirror of the formed cavity allows each region of the MoSe_2_ heterostructure to be coupled to the cavity modes independently^29^. Figure 2a shows a PL scan of a cavity formed with a concave mirror with a radius of curvature of 20 μm and a single monolayer MoSe_2_-active region. The various modes present in the spectra arise from the three-dimensional confinement of the photonic field, which gives rise to longitudinal modes and their associated higher-order transverse modes. These cavity modes are tuned through the neutral exciton resonance by reducing the mirror separation by applying a d.c. voltage to the bottom z-nanopositioner. The longitudinal resonance, labelled TEM_00_, is at an energy of 1.588 eV at *V*=0, and the modes at higher energy are its associated first (1.608 eV) and second (1.628 eV) transverse modes. The total optical cavity length is around 2.3 μm and the longitudinal mode number *q*=5. The modes at lower energy than the longitudinal mode are transverse modes associated with a different longitudinal mode at much lower energy (*q*−1). These are present since the mirror separation is larger than the separation of λ/4 required to reach the fundamental longitudinal resonance (*q*=1).

**Figure 3:**
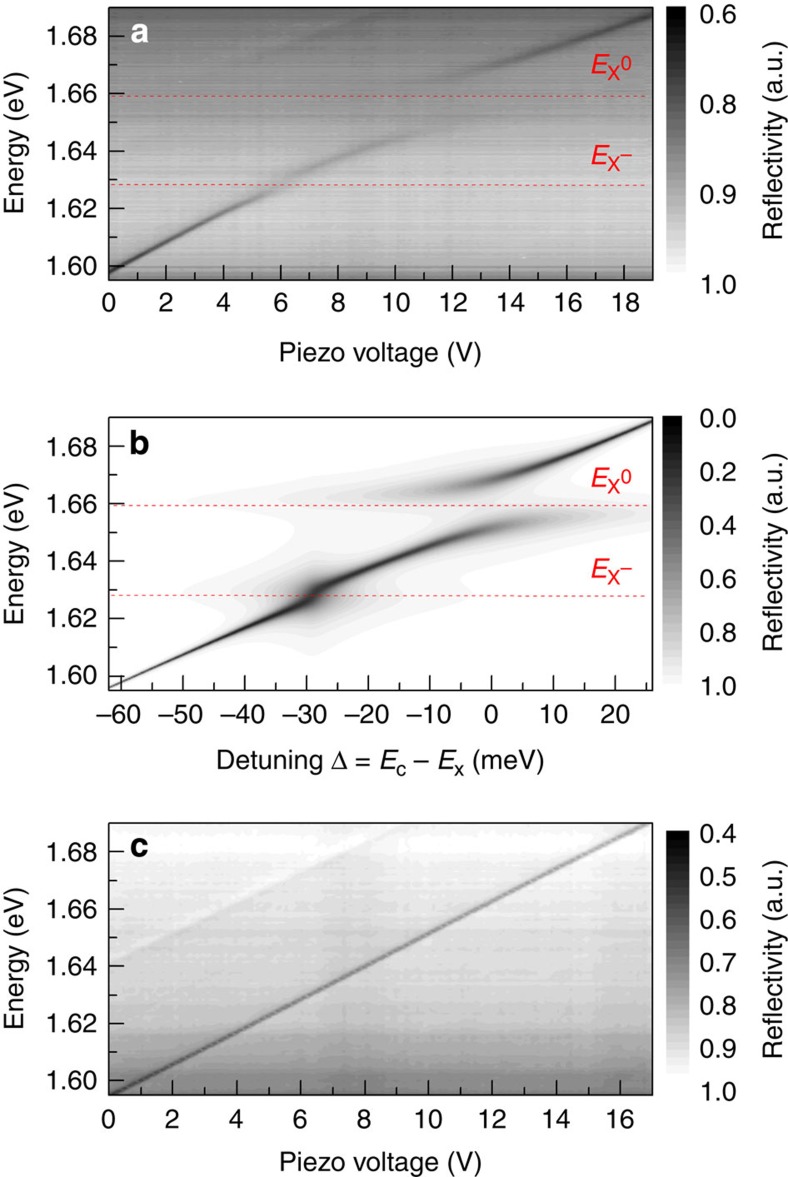
Reflectivity scans showing strong coupling for X^0^ and weak coupling for X^−^. (**a**) Reflectivity scan of a single-QW area at 4 K showing clear anticrossing with X^0^. Weak coupling with X^−^ is also apparent where the coupling strength is less than the charged exciton linewidth. (**b**) Theoretical reproduction of **a** based on a three-coupled oscillator model with coupling strengths of 18 and 8.2 meV for X^0^ and X^−^, respectively. (**c**) Reflectivity scan of an empty cavity with no active region showing a linear dependence of the cavity mode energy on the piezo voltage.

Clear anticrossings between the cavity mode resonances and the neutral exciton energy are observed revealing the formation of well-resolved polariton states. Each photonic mode is characterized by a specific field distribution in the plane of the TMDC layers and couples to an excitonic mode with the same in-plane distribution. The coherent absorption and emission of a photon by an exciton does not induce coupling between different photonic modes. Polariton states from different photon modes are therefore orthogonal and are well described by the coupling between a single photon mode and a single excitonic mode for each of them. A fit to the UPB and LPB peak energies for the longitudinal mode using a coupled oscillator model is shown by the dashed lines in Fig. 2b and reveals a Rabi splitting of *ℏ*Ω_Rabi_=20 meV for a single MoSe_2_ monolayer. Here we approximate the cavity mode energy as a linear function of applied voltage, which is supported by both transfer matrix simulations and reflectivity measurements (see Fig. 3c). The detuning is then defined as Δ=*E*_ph_−
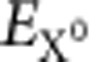
 where *E*_ph_ and 
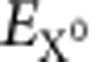
 are the fitted longitudinal cavity mode energy (TEM_00_) and the neutral exciton energy, respectively. Spectral slices of the PL at various detunings from Δ=−16 meV to Δ=+12 meV are displayed in Fig. 2c, where the LPB and UPB can be resolved. Moreover, the PL spectrum at zero detuning shown in Fig. 2d shows clearly two peaks where *ℏ*Ω_Rabi_ is significantly larger than the polariton linewidths *γ*_LPB_ and *γ*_UPB_.

The polariton linewidths are plotted as a function of longitudinal mode detuning in Fig. 2e. At large negative detunings of Δ<−30 meV, the LPB linewidth approaches the bare cavity linewidth of 0.8 meV due to the high photonic component of the polariton. At detunings of Δ=−30 to −20 meV, significant broadening of the LPB is observed, corresponding to resonance with the X^−^ energy. The UPB is observed at positive detunings up to the recorded detunings of Δ=+40 meV with a relatively narrow linewidth of around 2 meV.

Figure 3a shows a reflectivity scan of a single-QW area. Only the longitudinal mode is visible due to the poor mode matching between the Gaussian excitation spot and the lateral transverse mode profiles. As before, a clear anticrossing is observed when the mode is tuned through the neutral exciton energy. When the cavity mode is tuned through resonance with the X^−^ energy, a small shift in the cavity energy can be observed, in contrast to the broadening observed in PL. In Fig. 3b, we theoretically reproduce the experimental result shown in Fig. 3a based on a three-coupled oscillator model. We use the exciton linewidths from Fig. 1d and reproduce the coupling behaviour using coupling strengths for X^0^ and X^−^ of 18 and 8.2 meV, respectively. This is consistent with the reduced oscillator strength of X^−^ evidenced through its low absorption^39^.

**Figure 4:**
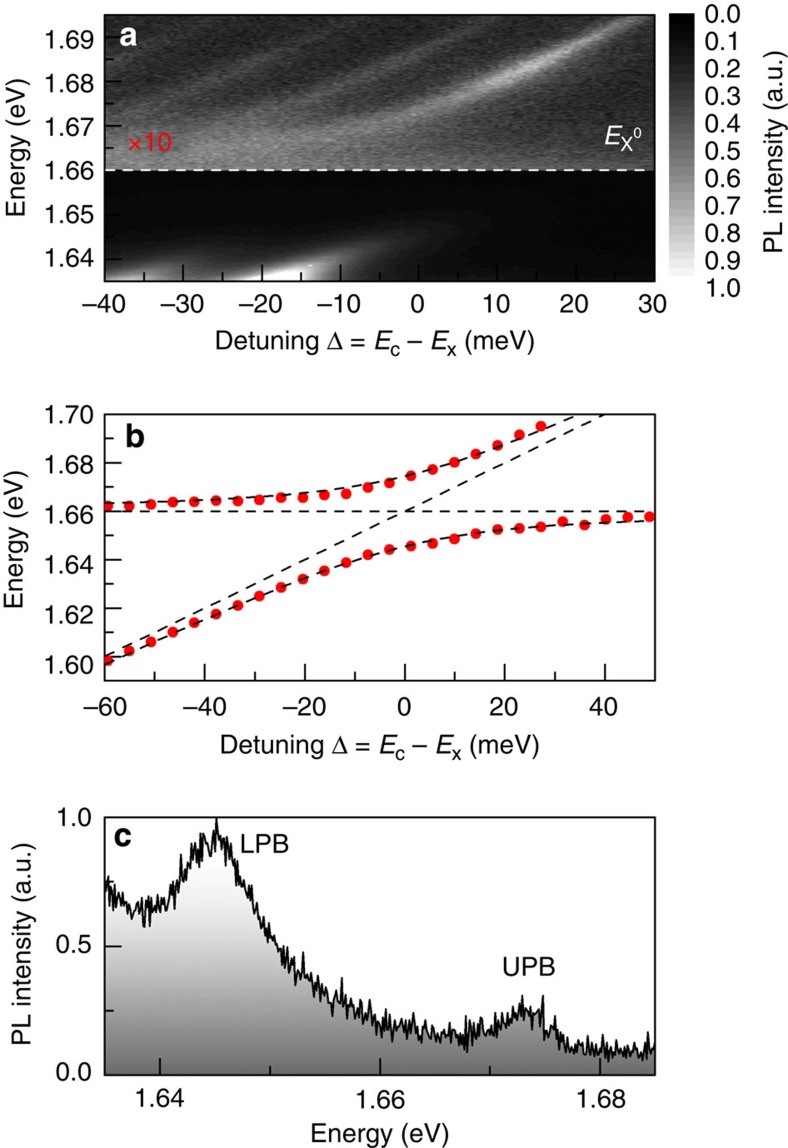
Observation of strong exciton–photon coupling in a MoSe_2_ double-QW heterostructure. (**a**) The double-QW structure shows an anticrossing between the neutral exciton and discrete cavity modes at 4.2 K. (**b**) A fit to the peak position as a function of detuning yields a Rabi splitting of 29 meV. (**c**) The upper and lower polariton branches are well resolved at resonance.

Figure 4a shows the PL spectra from a cavity formed with the double-QW region of the heterostructure as the active region. Here a clear anticrossing is observed between the cavity modes and neutral exciton, giving rise to well-resolved polariton branches at resonances (Fig. 4c). The extracted peak positions are plotted in Fig. 4b, where a fit to the polariton peak energies using the coupled oscillator model reveals an increased Rabi splitting of 29 meV. In contrast, as expected from the low oscillator strength of the optical transition, weak coupling is observed in a bilayer region of the sample (Supplementary Fig. 1), where MoSe_2_ has an indirect bandgap.

## Discussion

The extracted Rabi splitting of 20 meV for the single-QW region is in agreement with the theoretically calculated value of 26.7 meV (see Supplementary Note 1). Deviation in the theory from the experimentally measured value is most likely caused by inaccuracies in the estimation of the exciton wavefunction, as the electron and hole effective masses and exciton binding energy are not known accurately. Using the experimental value, we estimate the radiative lifetime of the neutral exciton of around *τ*=0.4 ps (see Supplementary Note 2), corresponding to a homogeneous linewidth of Γ_0_=1.6 meV in agreement with recent work in WSe_2_ monolayers^40^. This is around 13 × faster than the exciton lifetime of 5.3 ps measured in Supplementary Fig. 2, which is determined by relaxation to low k-states. The Rabi splitting for the double-QW region is 29 meV, in agreement with the expected scaling of the Rabi splitting with QW number 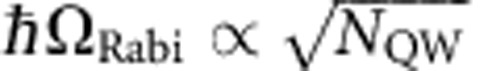 (ref. 41). We anticipate many applications of multiple-QW heterostructures where, due to the large exciton-binding energy, stable room-temperature polaritons can be expected. A heterostructure consisting of four MoSe_2_ QWs is expected to exhibit a Rabi splitting of at least 40 meV, larger than the room-temperature exciton linewidth of 35 meV (see Supplementary Fig. 3), potentially allowing room-temperature polariton formation.

Our analysis of the PL linewidth in Fig. 2e provides further insight in the properties of the polariton states. It shows that at zero exciton–photon detuning, the single-QW LPB linewidth of *γ*_LPB_=4.9 meV is less than the linewidth averaged value of (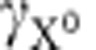
+*γ*_ph_)/2=5.9 meV predicted from the two-level coupled oscillator model, where *γ*_X0_ and *γ*_ph_ are the measured inhomogeneously broadened exciton linewidth and bare cavity linewidth, respectively, taken from PL measurements. This may be due to motional narrowing, which is expected in systems such as this where 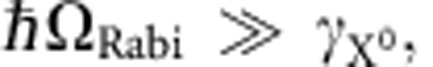 leading to averaging over the inhomogeneous broadening^42^. This causes the polariton linewidth to approach (
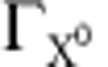
+*γ*_ph_)/2 where Γ_X0_ is the homogeneous neutral exciton linewidth^43^. Alternatively, this narrowing may be due to the reduced number of excitonic states that couple to the photonic mode within the 1-μm beam waist of the mode, which may have a smaller linewidth in comparison with the 5-μm spot measured in Fig. 1c due to disorder in the film. Further insight in the polariton properties may be gained from the measurements of PL intensity as a function of the detuning (see Supplementary Fig. 4).

We also observe bright and narrow UPB resonance in both the single- and double-QW structures (see Figs 2 and 3). Figure 2e shows that in the single-QW structure, the UPB linewidth at resonance is 8.7 meV due to broadening from relaxation through scattering to the uncoupled exciton states^44^. For larger detunings, the linewidth reduces to ≈2 meV. The presence of the UPB with an intensity comparable to the LPB is possible in TMDCs since the binding energy, 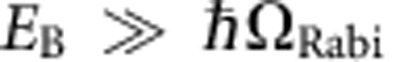 and hence the electron–hole continuum is far from the polariton resonances leading to much reduced relaxation of the UPB states. This can be quantified further through the ratio *ℏ*Ω_Rabi_/*E*_B_, which is around ≈0.04 for MoSe_2_ in contrast to >0.2 for all other materials where strong coupling was observed, as shown in Supplementary Table 1. The well-resolved UPB resonance also remains on the mirror stopband.

In addition to the behaviour due to the interaction with the X^0^ resonance described above, a strong broadening of the cavity PL is observed in resonance with X^−^ in Fig. 2e. We attribute this to the weak coupling between the X^−^ states and the cavity mode. Here the X^−^coupling strength is comparable to the corresponding polariton linewidths in PL, preventing the polariton branches from being resolved. However, in reflectivity (Fig. 3a) a clear modal shift is present when tuning across X^−^ instead of the broadening observed in PL. This difference in behaviour can be understood through the dependence of the observed Rabi splitting on the measurement method, such as absorption, reflectivity or transmission, when the polariton linewidths are comparable to the mode splitting. In this case, the splitting in reflectivity is expected to be larger than in PL (Ω_R_>Ω_PL_)^45^. The X^−^coupling strength is proportional to 
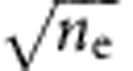
, where *n*_e_ is the electron density^46^ from inherent doping from impurities in the 2D film. Note that it has been shown in GaAs-based systems that the total oscillator strength of all excitonic components is a conserved quantity and hence, the presence of X^−^ due to doping may act to reduce the coupling strength of X^0^ (ref. 46).

In summary, we have conclusively demonstrated strong exciton–photon coupling of MoSe_2_ heterostructures in tunable optical microcavities through the observation of an anticrossing with the neutral exciton energy in PL and reflectivity. For a single MoSe_2_ monolayer, a Rabi splitting of 20 meV is observed for X^0^. We extended this to the demonstration of multiple-QW TMDC heterostructures in the strong-coupling regime where the Rabi splitting is increased to 29 meV. In single-well structures, we also observe the weak-coupling regime with the charged exciton. We estimate the coupling strength to be 8.2 meV, less than the charged exciton linewidth preventing charged polariton branches from being resolved in PL. Unique to TMDC microcavities is the presence of a bright and narrow UPB due to the large exciton-binding energy causing the electron–hole continuum to be far from the polariton resonance. This will allow the potential realization of phenomena involving the UPB, such as highly nonlinear parametric processes with balanced idler/signal, or polariton quantum bits^47^. Other avenues of VDW crystal-based polaritonics may include studies involving the spin–valley coupling between excitonic states, which leads to the formation of longitudinal-transverse polarized excitons with potential large polarization splitting that will be inherited by the polaritonic system^48^. This type of spin–orbit interaction is at the origin of the optical spin Hall effect^49^ and the formation of persistent spin currents in polariton condensates^50^. It allows manipulation of polariton trajectories through the emergence of non-Abelian gauge fields^51^. Lattices of coupled open cavities^52^ filled with VDW heterostructures could be realized in the near future and serve as a basis for the realization of polaritonic topological insulators^53^,^54^,^55^ with room-temperature operation.

From a technological point of view, the results presented here open the way to exploit VDW heterostructures in electrically pumped polariton devices. The external cavity geometry offers high flexibility in the use of complex heterostructures, which incorporate graphene contacts for vertical current injection, with the potential of room-temperature operation. Given the existing technological challenges with other promising materials such as GaAs^56^, GaN and ZnO, the potential of the VDW heterostructures for electrically pumped polariton devices appears to be high and of significant technological interest.

## Methods

### Dielectric mirror fabrication

The concave-shaped template for the top mirror is produced by focused ion beam milling, where an array of concave-shaped mirrors with radii of curvatures ranging from 7 to 20 μm is milled in a smooth fused silica substrate. Gallium ions are accelerated onto a precise position of the silica substrate achieving an accuracy of around 5 nm with an r.m.s. roughness below 1 nm (ref. 38). The highly reflecting DBRs are coated by ion-assisted electron beam deposition, where 10 pairs of quarter-lambda SiO_2_/NbO_2_ layers (refractive index 1.4 and 2.0, respectively), designed for a centre wavelength of 750 nm and a stop-bandwidth of around 200 nm, are deposited on the substrates.

### 2D film fabrication

The monolayer sheets of MoSe_2_ and the thin films of hBN were obtained by mechanical exfoliation of bulk crystals. The first monolayer is transferred onto a thin layer of hBN using standard transfer techniques^57^. This step is repeated with the 3-nm-thick sheet of hBN and the second monolayer sheet of MoSe_2_. The final heterostructure, consisting of a single monolayer region, a double-QW region and a MoSe_2_ bilayer region was then transferred onto the planar dielectric mirror. The individual regions can be identified using optical imaging. Bulk crystals were acquired from HQGraphene.

### Optical measurements

Optical measurements were performed with the samples placed in a helium bath cryostat system at a temperature of 4.2 K. Top and bottom mirrors were attached to attocube closed-loop XYZ nanopositioners and a tilt positioner allowing independent sample positioning with a travel range of 5 mm with a few tens of pm precision. The optical properties of the monolayer MoSe_2_ can be measured when the top mirror is moved out of the optical path using the lateral translation stages. A plano-concave microcavity is formed when the concave mirror is brought back into the optical path. All μ-PL experiments were performed with a continuous-wave excitation using a 638-nm laser diode, focused onto the sample using an achromatic lens. The collected PL is focused onto a wound fibre bundle and guided into a 0.75-m spectrometer and a high-sensitivity charge-coupled device for emission collection. Time-resolved measurements are obtained using a picosecond-pulsed, frequency-doubled titanium–sapphire laser with a pulse length of around 3 ps. The exponential decay of the monolayer emission is collected using a streak camera.

## Additional information

**How to cite this article:** Dufferwiel, S. *et al*. Exciton–polaritons in van der Waals' heterostructures embedded in tunable microcavities. *Nat. Commun.* 6:8579 doi: 10.1038/ncomms9579 (2015).

## Supplementary Material

Supplementary Information Supplementary Figures 1-4, Supplementary Table 1, Supplementary Notes 1-2 and Supplementary References
