# Supplementary material for: Exciton–polaritons in van der Waals heterostructures embedded in tunable microcavities
Source: Nat Commun. 2015 Oct 8;6:8579. doi: 10.1038/ncomms9579 (PMC4633950; doi:10.1038/ncomms9579)
Supplement: Supplementary Information — Supplementary Figures 1-4, Supplementary Table 1, Supplementary Notes 1-2 and Supplementary References [file ncomms9579-s1.pdf]

SUPPLEMENTARY FIGURES

Supplementary Figure 1: Studies on bilayer molybdenum diselenide sheets

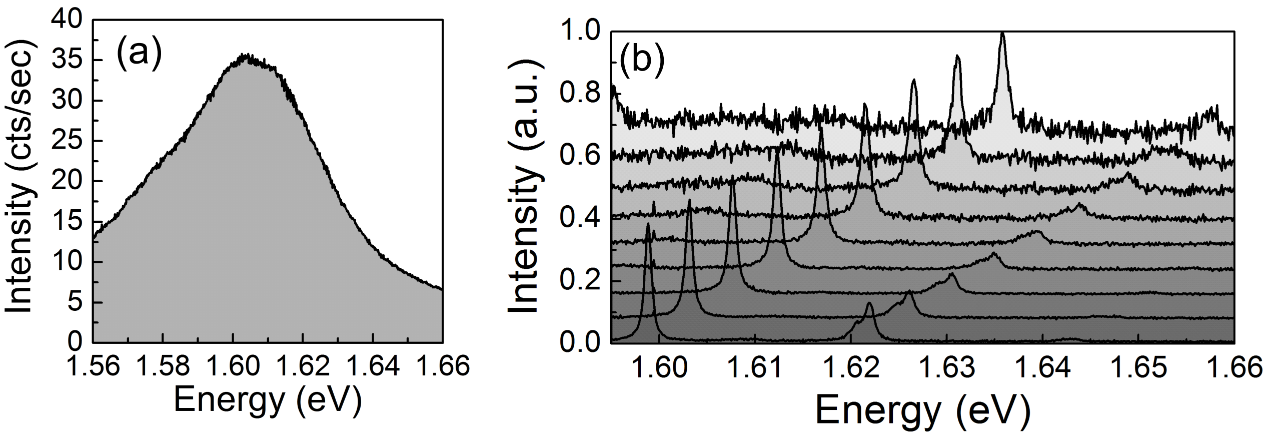

FIG. 1. **Crossing between the cavity modes and $MoSe_2$ bilayer heterostructure area.** (a) Bilayer PL emission due to indirect transition at 4.2 K. PL is measured from the bilayer area marked by the black border in Figure 1(c) in the main text. (b) Spectra of the cavity emission with a bilayer active region showing weak coupling. The band-structure of bilayer $MoSe_2$ shows that there is an indirect transition causing a reduction in the emission efficiency. When the bilayer is coupled to the cavity modes, weak coupling is observed as shown in (b) where a crossing through the exciton resonance is observed.

Supplementary Figure 2: Exciton Dynamics

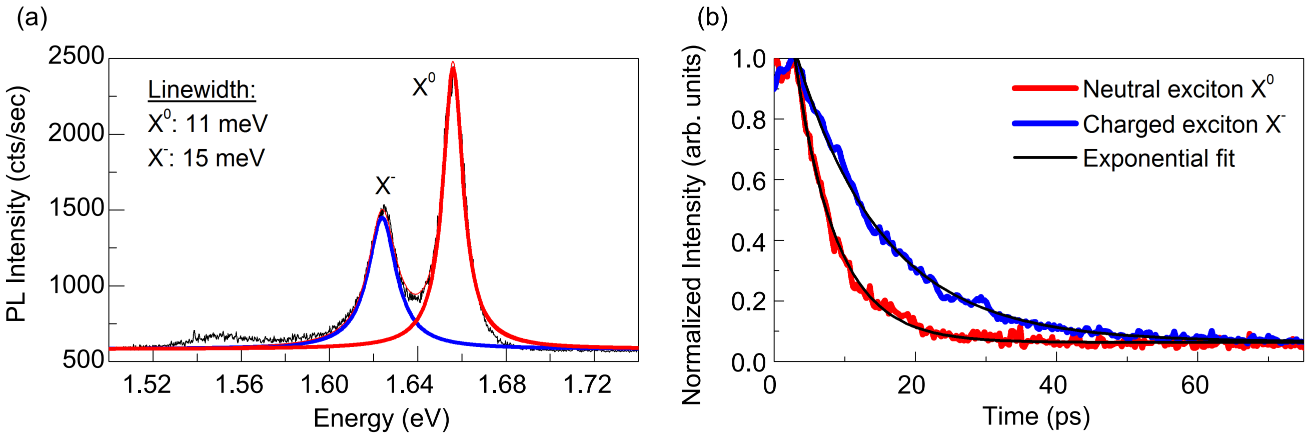

FIG. 2. **Photoluminescence of a $MoSe_2$ monolayer.** (a) The PL emission of a monolayer of MoSe2 at 4.2 K shows two characteristic peaks attributed to a neutral (X^0) and charged (X^-) exciton with a measured linewidth of 11 meV for X^0 and 15 meV for X^-. (b) Time-resolved measurements reveal a PL lifetime of 5.3 ps for X^0 and 12.5 ps for X^-.

Supplementary Figure 3: Room temperature measurements

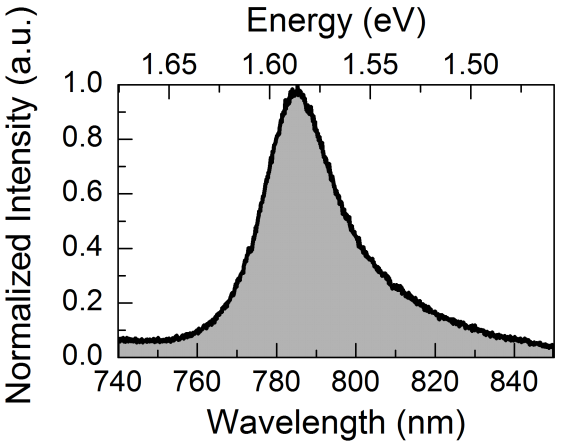

FIG. 3. **Room temperature PL of a $MoSe_2$ monolayer.** A single broad resonance of X^0 is observed at room temperature. The PL spectrum of X^0 in a $MoSe_2$ monolayer at room temperature shows a linewidth of around 35 meV, exceeding the vacuum Rabi splitting of both the single and double QW heterostructure. Therefore weak coupling is observed at room temperature. The demonstrated dependence of $\Omega_{rabi} \propto \sqrt{N_{QW}}$ indicates that a heterostructure consisting of four or more $MoSe_2$ QWs will increase the Rabi splitting sufficiently to resolve both the UPB and LPB at room temperature.

Supplementary Figure 4: Polariton intensity

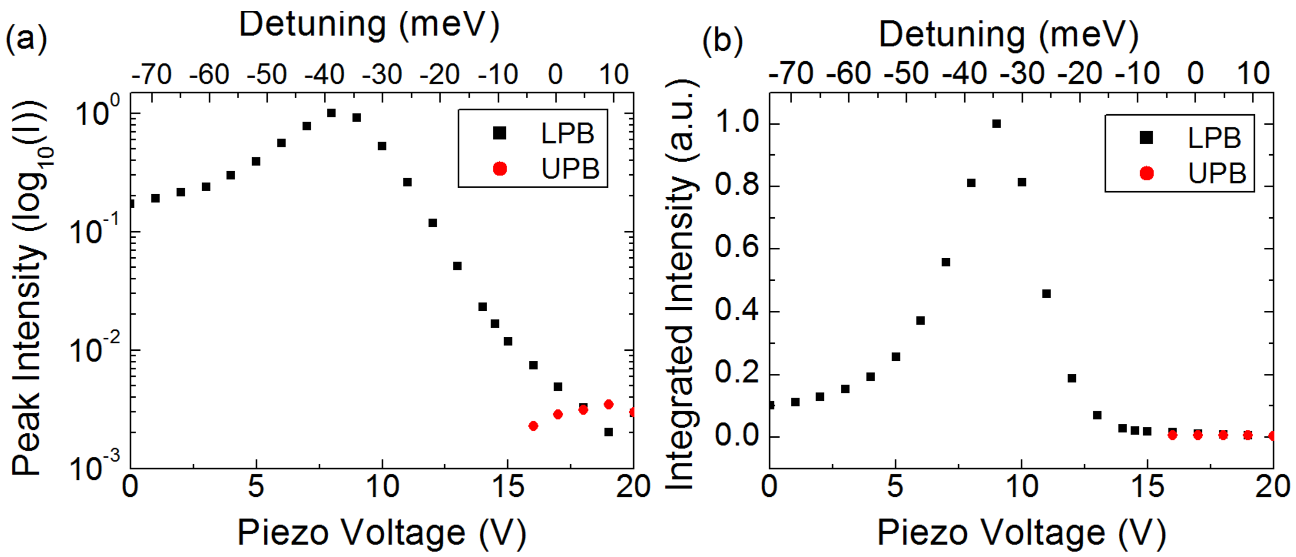

FIG. 4. **Polariton intensity.** (a) Peak intensity of the polariton resonance as a function of the piezo voltage. The detuning is estimated from the coupled oscillator model fit in Figure 2 of the main text. (b) Integrated intensity as a function of the piezo voltage and cavity-mode-X^0 detuning. The maximum in the peak intensity in (a) is achieved slightly below the X^- energy ($\delta = -35$ meV), where the peak intensity is 2 orders of magnitude larger than when in resonance with X^0. The integrated intensity in (b) shows a significant integrated PL enhancement around $\delta = -35$ meV, a few meV below X^-. This detuning dependence of the PL is due to the interplay between energy relaxation and non-radiative decay of excitons and polaritons as well as the cavity pulling effect[1]. These effects are material- and sample-dependent. A fuller understanding of these phenomena will require a separate investigation of $MoSe_2$ heterostructures in order to understand the exciton-phonon, exciton-exciton and exciton-free-carrier interactions, and also behaviour of low-energy localised states which vary from sample to sample.

SUPPLEMENTARY TABLES

Supplementary Table 1: Comparison of various material systems in which strong coupling has been observed

Material	Binding energy in bulk (QWs) (E_B)	Rabi splitting (Ω_{Rabi})	Ω_{Rabi}/E_B	Polariton linewidth at resonance
GaAs	4.8 ($\sim$ 14) meV[2,3]	$\sim$ 3-15 meV[4,5]	$\sim$ 0.2-1.1	$\sim$ 0.1-1 meV
CdTe	10 ($\sim$ 25) meV[3,6]	$\sim$ 16-26 meV[6,7]	$\sim$ 0.6-1	$\sim$ 0.6 meV[7]
GaN	26 ($\sim$ 40) meV[8,9]	$\sim$ 50 meV[9]	$\sim$ 1	$\sim$ 10-15 meV[10,11]
ZnO	60 meV[12]	$\sim$ 200 meV[13]	$\sim$ 3	$\sim$ 1.5-10 meV[13]
Organics	250 - 500 meV[14]<br>1 eV[15]	$\sim$ 110 meV[14]<br>$\sim$ 200 meV[15]	$\sim$ 0.2-0.5	$\sim$ 16.5-25 meV[14,15]
$MoSe_2$	470 meV[16]	20-29 meV	$\sim$ 0.04	$\sim$ 4-9 meV

SUPPLEMENTARY NOTES

Supplementary Note 1: Theoretical calculation of the expected Rabi splitting

Coupling a photonic open cavity mode to an exciton level is characterized by the Rabi frequency[17]:

$$\Omega_{Rabi} = 2\sqrt{\frac{2\Gamma_0 c}{n_c(L_{DBR}+L_c)}}, \tag{1}$$

where n_c is the cavity refractive index, which is close to unity in the open cavity system, L_{DBR} and L_c are the effective mirror and cavity length length respectively. $\Gamma_0 = 1/2\tau$ is the exciton radiative broadening given by[18]:

$$\Gamma_0 = \frac{2\pi e^2 |p_{cv}|^2}{nc\hbar\omega_0 m_e^2}\phi(\rho)^2, \tag{2}$$

where e is the electron charge absolute value, p_{cv} is the matrix element of the momentum between electron Bloch functions at valence and conduction band edges, $n \approx 2.2$ is the refractive index of $MoSe_2$, c is the speed of light, $\hbar\omega_0$ is the cavity mode energy, m_e is the free electron mass and $\phi(\rho)$ is the internal motion part of the 2D exciton wavefunction. For the 1s exciton state one can write:

$$\phi(0) = \sqrt{\frac{2}{\pi a_B^2}} = \sqrt{\frac{2\mu E_b}{\pi\hbar^2}}, \tag{3}$$

with a_B the 2D Bohr radius, $E_b \approx 0.55$ eV the exciton binding energy and $\mu = m_e\frac{m_c^* m_v^*}{m_c^*+m_v^*}$ the exciton reduced mass.

The matrix element p_{cv} may be deduced from the electron effective mass expression given by the $k \cdot p$ method:

$$m_c^* = \frac{m_c}{m_e} = \left(1+\frac{2|p_{cv}|^2}{E_g m_e}\right)^{-1}, \tag{4}$$

where $E_g \approx 2.1$ eV is the band gap. Conduction and valence band effective masses are calculated ab initio for $MoSe_2$ and are given by $m^*_{c(v)} = 0.70(0.55)$[19]. From this we derive:

$$|p_{cv}| = \sqrt{\frac{E_g m_e}{2}\left(\frac{1}{m_c^*}-1\right)}. \tag{5}$$

Substituting equations 2 and 5 in equation 1 and assuming no detuning between exciton energy and photonic mode ($\hbar\omega_0 = E_g - E_b$) we obtain the Rabi splitting:

$$\hbar\Omega_{Rabi} = 8\sqrt{\frac{m_v^*(1-m_c^*)}{2n(m_c^*+m_v^*)}\frac{E_g E_b}{E_g-E_b}\frac{e^2}{L_{DBR}+L_c}}. \tag{6}$$

With the absolute effective cavity length $L_{DBR}+L_c = 2.3$ μm , the obtained Rabi splitting is $\hbar\Omega_{Rabi} \approx 26.7$ meV. This agrees well with the experimentally obtained value of 20 meV.

Supplementary Note 2: Calculation of the exciton radiative lifetime

The obtained Rabi splitting for a single monolayer sheet is $\Omega_{Rabi} = 20$ meV. Following equation 1, the exciton radiative rate Γ_0 and therefore the radiative lifetime τ can be obtained using

$$\Omega_{Rabi} = \frac{20 \text{ meV}}{\hbar} = 2\sqrt{\frac{2\Gamma_0 c}{n_c(L_{DBR} + L_c)}}. \tag{7}$$

The total cavity length is determined by the free spectral range between two longitudinal modes $(L_{DBR}+L_c) = 2.3$ μm, c is speed of light and $n_c = 1.4$ is the effective cavity refractive index. This allows the exciton radiative lifetime to be calculated to be $\Gamma_0 = \frac{1}{0.8 \text{ ps}}$. With $\Gamma_0 = \frac{1}{2\tau}$ the exciton radiative lifetime is then $\tau = 0.4$ ps. This is around 13x faster than the exciton lifetime of 5.3 ps measured in Supplementary Figure 1 which is determined by relaxation effects to low k-states. The homogeneous exciton linewdith is then given by $\Delta E = \hbar/\tau = 1.6$ meV. This is much smaller than the low temperature PL linewidth of 11 meV indicating that significant broadening occurs due to disorder effects, an aspect which might be improved using epitaxial layers allowing much narrower polariton linewidths.

SUPPLEMENTARY REFERENCES

[1] Stanley, R. P. *et al.* Cavity-polariton photoluminescence in semiconductor microcavities: Experimental evidence, *Phys. Rev. B* **53** 10995-11007 (1996)

[2] Atanasov, R. *et al.* Exciton properties and optical response in $In_xGa_{1-x}As/GaAs$ strained quantum wells, *Phys. Rev. B* **50** 14381-14388 (1994)

[3] Yu, P. Y., Cardona, M. Fundamentals of Semiconductors: Physics and Materials Properties, *Springer, Berlin* (2005)

[4] Weisbuch, C. *et al.* Observation of the coupled exciton-photon mode splitting in a semiconductor quantum microcavity, *Phys. Rev. Lett.* **69** 3314-3317 (1992)

[5] Wertz, E. *et al.* Spontaneous formation and optical manipulation of extended polariton condensates, *Nature Physics* **6** 860-864 (2010)

[6] Andre, R. *et al.* Spectroscopy of polaritons in CdTe-based microcavities, *J. of Crystal Growth* **184** 758-762 (1998)

[7] Kasprzak, J. *et al.* BoseEinstein condensation of exciton polaritons, *Nature* **443** 409-414 (2006)

[8] Kornitzer, K. *et al.* Photoluminescence and reflectance spectroscopy of excitonic transitions in high-quality homoepitaxial GaN films, *Phys. Rev. B* **60** 1471-1473 (1999)

[9] Christmann, G. *et al.* Large vacuum Rabi splitting in a multiple quantum well GaN-based microcavity in the strong-coupling regime, *Phys. Rev. B* **77** 085310 (2008)

[10] Christmann, G. *et al.* Room temperature polariton lasing in a GaN/AlGaN multiple quantum well microcavity, *Appl. Phys. Lett.* **93** 051102 (2008)

[11] Christopoulos, S. *et al.* Room-Temperature Polariton Lasing in Semiconductor Microcavities, *Phys. Rev. Lett.* **98** 126405 (2007)

[12] Klingshirn, C. F. *et al.* Zinc oxide: from fundamental properties towards novel applications, *Springer* (2010)

[13] Li, F. *et al.* From Excitonic to Photonic Polariton Condensate in a ZnO-Based Microcavity, *Phys. Rev. Lett.* **110** 196406 (2013)

[14] Lidzey, D. G. *et al.* Strong excitonphoton coupling in an organic semiconductor microcavity, *Nature* **395** 53-55 (1998)

[15] Kena-Cohen, S., Forrest, S. R. Room-temperature polariton lasing in an organic single-crystal microcavity, *Nature Photonics* **4** 371-375 (2010)

[16] Berkelbach, T. C., Hybertsen, M. S., Reichman, D. R. Theory of neutral and charged excitons in monolayer transition metal dichalcogenides, *Phys. Rev. B* **88** 045318 (2013)

[17] Kavokin, A. V. *et al.* Microcavities, Series on Semiconductor Science and Technology, *Oxford University Press* (2007)

[18] Ivchenko, E. L. *et al.* Optical spectroscopy of semiconductor nanostructures, *Alpha Science International Ltd* (2007)

[19] Nalitov, A. V. *et al.* Spin-Orbit Coupling and the Optical Spin Hall Effect in Photonic Graphene, *Phys. Rev. Lett.* **114** 026803 (2015)
